# Starting School Healthy and Ready to Learn: Using Social Indicators to Improve School Readiness in Los Angeles County

**Published:** 2007-09-15

**Authors:** Cheryl Wold, Will Nicholas

**Affiliations:** Wold and Associates. Ms. Wold was formerly Chief of the Health Assessment Unit in the Los Angeles County Department of Health Services—Public Health.; The California Endowment, Los Angeles, California. Dr. Nicholas was formerly Senior Research Analyst at First 5 Los Angeles, Los Angeles, California

## Abstract

**Background:**

School readiness is an important public health outcome, determined by a set of interdependent health and developmental trajectories and influenced by a child's family, school, and community environments. The same factors that influence school readiness also influence educational success and health throughout life.

**Context:**

A California cigarette tax ballot initiative (Proposition 10) created new resources for children aged 0 to 5 years and their families statewide through county-level First 5 commissions, including First 5 LA in Los Angeles County. An opportunity to define and promote school readiness indicators was facilitated by collaborative relationships with a strong emphasis on data among First 5 LA, the Children's Planning Council, and the Los Angeles County Public Health Department, and other child-serving organizations.

**Methods:**

A workgroup developed school readiness goals and indicators based on recommendations of the National Education Goals Panel and five key domains of child well-being: 1) good health, 2) safety and survival, 3) economic well-being, 4) social and emotional well-being, and 5) education/workforce readiness.

**Consequences:**

The Los Angeles County Board of Supervisors and First 5 LA Commission adopted the school readiness indicators. First 5 LA incorporated the indicators into the results-based accountability framework for its strategic plan and developed a community-oriented report designed to educate and spur school readiness-oriented action. The Los Angeles County Board of Supervisors approved a countywide consensus-building plan designed to engage key stakeholders in the use of the indicators for planning, evaluation, and community-building activities.

**Interpretation:**

School readiness indicators in Los Angeles County represent an important step forward for public health practice, namely, the successful blending of an expanded role for assessment with the ecological model.

## Background

Beginning school healthy and ready to learn is greatly influenced by the first 5 years of life and, in turn, influences health throughout life. During the prenatal period through age 5, biology, social relationships, and environments interact "continuously and dynamically" to profoundly influence future health and well-being (1). Prenatal and early life exposures to environmental toxins (e.g., lead, pollution), substance abuse, and chronic economic and social stress (e.g., poverty, parental depression, violence) can have profound effects throughout childhood and adulthood. Life-course analyses have demonstrated that many common disorders have modifiable origins early in childhood (2,3). Genetic predisposition and social and environmental factors interact with adaptive responses to influence later health, including life expectancy, the development of chronic diseases, and reduced functioning (4-7). Furthermore, optimal health development in children is achieved through nurturing, safe interactions and experiences with families and caregivers in the context of health-sustaining community environments. School readiness is therefore an important outcome that reflects health and developmental influences early in life but also reaches far throughout the life course (8) as the numerous positive health, social, and economic benefits associated with educational attainment are conferred (9,10). 

Indicators are powerful tools to support planning, community engagement, policy, and advocacy on behalf of children and families. Indicators have been used successfully to promote accountability among governmental and nongovernmental agencies and to engage partners in civic efforts (11,12). Indeed, improved data quality and accessibility at the national and state levels during the past 2 decades have prompted the widespread development of report cards and indicators to gauge progress in public health and other social, environmental, and economic areas (13). Public health departments can play a vital role, whether as generators of data, stakeholders, or conveners, in the use of indicators to drive changes in community well-being (14).

Within the field of early child development, a movement has emerged to track the dynamics of multiple influences on early childhood health and developmental trajectories through the evolved concept of school readiness. The 1992 National Education Goals Panel (NEGP) recommendations were instrumental in recognizing the essential contextual influences — family, schools, and communities — on school readiness (15). These recommendations stood in sharp contrast to outdated concepts of school readiness that were previously associated with the assessment of a child's maturity and cognitive development as a qualification for school entry (8).

Capitalizing on this movement, Los Angeles (LA) County developed a set of school readiness indicators as a tool for engaging community, monitoring trends, and implementing a results-based accountability framework for a local funding agency. This community case study describes the development, implementation, and early results from the experience.

## Context

Despite some positive gains, wide and persistent disparities remain in several indicators of child well-being in LA County. Attempts to align early education, health, and social service systems around common outcomes have faced many challenges, including the fragmentation of services brought on by funding stream-induced silos and the dominance of a programs and services mindset over more holistic population-based approaches. Other, more positive contextual factors contributed to the successful launch of school readiness indicators in LA County.

First, an outcomes-focused children's agenda emerged during the 1990s in LA County, guided by the use of data to drive changes in the systems serving children and families. A few prominent institutions invested in the development and dissemination of high-quality data (16,17). Results-based accountability and performance management practices, initiated through the sponsorship of trainings by lead county agencies, shifted the foci among institutions that serve children toward getting the most out of dollars invested (18) and showing measurable results (19).

Second, historically isolated departments had begun working more closely together. The Children's Planning Council (CPC; www.childrensplanningcouncil.org)Second, historically isolated departments had begun working more closely together. The Children's Planning Council (CPC; www.childrensplanningcouncil.org), established in 1991, actively began promoting better interdepartmental and public–private coordination of resources for improving conditions and services for children and families in the county. Working with other public and private institutions, the CPC was instrumental in shaping an outcomes-based agenda. Among its contributions were the promotion of five countywide outcomes for children: 1) good health, 2) safety and survival, 3) economic well-being, 4) social and emotional well-being, and 5) education/workforce readiness, and the creation of eight geographic subregions in the county called service planning areas (SPAs).

The third important contextual factor was the active involvement of LA County's Department of Public Health (DPH), which had made significant investments in local data development and dissemination. Central to those efforts was the 1997 launch of the Los Angeles County Health Survey, a biennial population-based telephone survey of more than 8000 adults and 6000 children. Critical gaps in local early childhood have been addressed by an extensive section devoted to children aged 0 to 5 years that asks about health and development, family and home environments, key parenting practices (e.g., reading to child, breastfeeding) and perceptions, access to childcare, and barriers to preventive health care (20). Indicators development work by The University of California, Los Angeles's (UCLA's) Center for Healthier Children, Families, and Communities had helped to push forward the value of indicators focused on social and environmental as well as life course determinants, which greatly influenced the data collected by the survey. The value of similar data on conditions and practices at home and in the community has been demonstrated nationally by the National Survey of Early Childhood Health Commonwealth Survey, National Survey of American Families (21,22).

The final precipitating factor was the passage in 1999 of Proposition 10, a statewide ballot initiative that levied a cigarette tax of 50 cents per pack to fund programs, policies, and systems improvements targeting children aged 0 to 5 years and their families statewide, through local county "First 5 Commissions." The Los Angeles County Commission (First 5 LA) has heightened attention to the first 5 years of life and has adopted school readiness as an overarching goal. In keeping with its results-based accountability approach to funding (12), First 5 LA sought to develop a core set of school readiness indicators to guide its evaluation efforts across its three investment areas: 1) health, 2) early learning, and 3) safety.

## Methods

The Los Angeles County School Readiness Indicator (SRI) Workgroup was convened in January 2003 to develop goals and related indicators with three objectives. The first was to *engage* the many agencies and individuals working with young children and families in communities throughout the county. Indicators that could be easily communicated and understood would provide a common language to support a dialogue about the actions needed by parents and families, child care providers, school personnel, politicians, and all citizens, to improve school readiness and school success. Second, the indicators would provide a *results-based accountability* framework for First 5 LA and partnering organizations to better align resources and action toward common school readiness goals. Third, the indicators would provide a tool for *monitoring trends *in conditions for school readiness over time.

The framework and criteria for indicator selection developed by the workgroup (Figure 1) highlighted the importance of moving beyond the abilities of children to capture the influences of family, community, and school environments and reflect both systemic and population-level indicators. The framework relied on the National Education Goals Panel's (NEGP's) working definition of school readiness: children's readiness for school, school's readiness for children, family and community supports and services that contribute to children's readiness for school success. It also related to the five outcomes of child well-being adopted by the county.

**Figure 1:** Framework and criteria for School Readiness Indicator Workgroup, Los Angeles County, 2003.

The following criteria were developed to provide principles and a framework for the indicator development. The list of indicators was to be concise (i.e., approximately 10 in number), practical (i.e., actionable), and strategic (i.e., linked to realistic local opportunities).
The indicators would be chosen to track school readiness in the following contexts:
Children ready for school Schools ready for children Families supporting children Communities supporting families and children
The indicators were to reflect the five outcomes adopted by the Board of Supervisors in 1993:
Good health Safety and survival Economic well-being Social and emotional well-being Education/workforce readiness
Indicators will be selected because they are understandable, the data are of high quality, and they measure an important aspect of school readiness. The ideal indicators are those with high "communication power" (i.e., understandable to a broad audience), "data power" (i.e., data are regularly collected and are of high quality), and "proxy power" (i.e., they are a reasonable proxy measure for, and reflect some important aspect of, school readiness) (12).

School readiness goals deemed important by the workgroup would be included in the final indicator set regardless of the availability of ideal data. A data development agenda was developed to encourage future work on indicators for these hard-to-measure goals. For example, "children are born at healthy birth weights" relies on data from birth records, and "families have adequate food" relies on survey data collected using a food insecurity measure. However, "schools, families, and caregivers work together to ensure a positive transition to K through 6 education" lacks a data source that met the selection criteria. Since the transition to school is an important component of school readiness, this goal was included without a corresponding indicator to encourage the development of ways to measure this important construct (Figure 2). First 5 LA's report *Shaping the Future *includes a complete description of indicators and data sources (23). 

**Figure 2:** School readiness goals and indicators, Los Angeles County, 2003.

**Goals**	**Indicators**
Children are born at healthy birth weights.	Newborns with low and very low birthweights
Children receive preventive health care.	Children aged 0 to 5 years whose parents report having a regular source of health care; children aged 0 to 5 years who have health insurance; hospitalizations of children with asthma.
Children are free from abuse and neglect and thrive in permanent homes.	Child abuse and neglect reports to the Department of Child and Family Services that result in Emergency Response services for children aged 0 to 5 years.
Families ensure that children are safe from unintentional injuries.	To be developed.
Communities offer safe places for children to live and play.	Children aged 1-5 years whose parents say they can easily get to a park, playground, or other safe place to play.
Families have adequate food.	Households below 300% of the federal poverty guidelines and with dependents aged 18 or younger who are food insecure.
Families have adequate financial resources.	Children aged 0 to 5 years living in families with incomes below 200% of the federal poverty guidelines.
Communities offer affordable housing for families.	To be developed.
Families have supportive networks and are able to find information and assistance.	Children aged 0 to 5 years whose parents say it is "very" or "somewhat" easy to find someone to talk to when they need advice about raising their child.
Families have access to quality child care.	Children aged 0 to 5 years whose parents report difficulty finding the child care they need on a regular basis; licensed child care spaces for children aged 0 to 5 years.
Communities encourage educational attainment for families.	Infants born annually to women/men aged 21 years and older with at least 12 years of education.
Families and caregivers interact with children in ways that promote cognitive, linguistic, social–emotional, and physical development.	Children aged 0 to 5 years who are read to daily by a parent or family member.
Schools and child care programs promote an environment that is conducive to learning.	To be developed.
Schools, families, and caregivers work together to ensure a positive transition to K-6 education.	To be developed.
Communities support families and children with special needs.	Children aged 3 and 4 years who are identified with serious but often missed disabilities and are enrolled in special education programs.

## Consequences

### Community and stakeholder engagement

The LA County Board of Supervisors adopted the School Readiness Goals and Indicators (SRIs) and approved a countywide consensus building plan designed to engage key stakeholders in the use of the indicators for planning, evaluation, and community strengthening activities. To implement this plan, the CPC Service Planning Area Councils (SPACs) focused a large part of their community engagement efforts on school readiness. One council held a series of school readiness community forums, in which the indicators were used as a call to action for families, communities, and schools to do their part in ensuring children’s readiness for school. Parents organized themselves around specific actions they could take to promote the school readiness of children in their communities. Actions included 1) more intentional use of parent-child together time for learning purposes (e.g., reading labels at grocery store, measuring ingredients in the kitchen), 2) communicating with teachers and school administrators about ways to make the school environment more welcoming and engaging for parents, 3) working collectively through Neighborhood Action Councils to address neighborhood safety hazards (e.g., freeway on-ramps, unsanitary conditions).

The Los Angeles County Office of Education (LACOE) developed a school readiness action plan that aligns its Head Start goals and objectives with the SRIs. Also in keeping with the SRIs, LACOE has integrated a new social–emotional competence strand into its training curriculum for Head Start parents, along with technical assistance to parents to support a seamless transition of children from Head Start to the public school system. The Los Angeles Unified School District (LAUSD) has convened meetings with early education administrators and parents through its Parent Leadership Institute to educate them about the indicators and elicit feedback on their effective use. LAUSD has also incorporated many of the SRIs into its early education improvement plan and has developed performance measures based on the indicators.

Finally, as part of its SRI dissemination efforts, First 5 LA developed a tool that has supported these consensus building and community engagement activities. *Shaping the Future *(23), a community-oriented tool designed to promote school readiness, presents the indicator data in a user-friendly format designed to educate readers on the multifaceted nature of school readiness, provide a quick reference to all the school readiness goals and indicators, and suggest ways that communities can take specific action to improve performance on each of the indicators.

### Results-based accountability: from engagement to action

Incorporating the SRIs into First 5 LA's strategic plan and results-based accountability framework was a key step that ensured that the Commission's strategic efforts and funded grants would be guided by the holistic concept of school readiness. The strategic plan laid out three goal areas: 1) health, 2) early learning, and 3) safe children and families. The SRIs most relevant to each goal area were the outcome that the corresponding strategies would seek to effect. The progress of funded initiatives under each goal area would be tracked using performance measures linked to broader changes in population-level SRIs based on the best available research evidence. For example, funded grantees and partners in the Healthy Births Initiative (health goal area) are using performance measures to improve the quality of both prenatal care and comprehensive case management services for at-risk pregnant women with the ultimate goal of reducing poor birth outcomes (SR goal 1). Another important example is the Los Angeles Universal Preschool Initiative (early learning goal area), which is implementing a quality rating system for its subsidized child care slots toward the goal of increased access to quality child care (SR goal 10). One of the challenges encountered by First 5 LA in implementing a results-based accountability framework was how to focus on enough of the SRIs to address the full spectrum of school readiness while at the same time not diluting its efforts by trying to address too many of the indicators.

### Monitoring trends

In addition to using the SRIs to guide its funding priorities and the strategies of its grantees and partners toward measurable results, First 5 LA, its research partners (including DPH), and other collaborators have committed to monitoring trends in the SRIs with plans to analyze and disseminate the results every 2 years. The reliance on data from cross-sectional population and administrative sources, and the limitations and bias inherent in such sources, presents a challenge to monitoring the SRIs. For example, changes in how the indicator data are collected could appear as changes in trends or mask important trends when examined over time. Data can also become unavailable due to losses in funding. The SRIs, while not a perfect surveillance tool, provide valuable data and a focus on desired outcomes, which can then be logically linked to programmatic activities and performance measures.

## Interpretation

LA County's positive experience with the SRIs builds on two foundations of public health practice, namely, the core assessment function and practice based on the ecological model. Developing and tracking indicators of school readiness expands the core public health function of assessment — monitoring the health status of populations to identify and address emerging health issues (24) — by collecting and leveraging information to improve health. Notably, the assessment function of the indicators has been promoted at both the county government and grassroots levels as a strategy for more effective leveraging of change.

The NEGP definition of school readiness adopted by the LA County School Readiness Indicators Workgroup represents an ecological perspective on early childhood development and well-being. It includes characteristics of the child and those of the family, community, and school environment that are critical to school readiness at kindergarten entry. Although few public health practitioners would dispute the validity of the ecological model, fostering the cross-sector collaboration necessary to address the multiple layers of the model can be challenging. The case of school readiness is unique in that the concept originated in the early childhood education field but has been studied from a public health perspective as well (1). In LA County, the catalyst for bringing together the multidisciplinary SRI workgroup was the presence of the First 5 LA Commission, a public entity explicitly designed to develop and test the ecological perspective in the early childhood field along with organizations such as the CPC and the DPH, which have historically promoted and valued that model.

The biggest challenge in using the indicators as an accountability tool has been maintaining a clear distinction between contribution and attribution. Tracking indicators and measurable objectives accounts for the contribution of First 5 LA programs toward improving outcomes and in turn helps improve program operations. Attributing impacts to specific funding initiatives requires controlled evaluation methodologies not often feasible in dynamic community settings without valid comparison groups.

Early childhood initiatives have laid the groundwork for similar efforts in other geographic areas (25,26). In Los Angeles we have learned that, given the support of multiple sectors, the ability to leverage local data collection efforts, and a commitment to grass-roots community engagement, social indicators can be a unifying component of a cross-sector focus on supporting positive environments for children during their critical early years.
